# Imaging Features of Morel-Lavallée Lesions

**DOI:** 10.5334/jbr-btr.1401

**Published:** 2017-12-16

**Authors:** Tineke De Coninck, Filip Vanhoenacker, Koenraad Verstraete

**Affiliations:** 1 Ghent University Hospital, BE; 2 AZ Sint Maarten, Duffel, BE

**Keywords:** Morel-Lavallée, Seroma, Hematoma, MRI, Ultrasound

## Abstract

**Objectives:**

To review the imaging characteristics of Morel-Lavallée lesions with both ultrasound and magnetic resonance imaging (MRI).

**Materials and Methods:**

We retrospectively analyzed 31 patients (mean age = 46 years), diagnosed with a Morel-Lavallée lesion, on ultrasound (n = 15) or MRI (n = 16). On ultrasound the echogenicity, internal septations, hyperechoic fat globules, compressibility and Doppler signal were evaluated. On MRI, T1- and T2-signal intensity, capsule presence, internal septations, enhancement, mass-effect and fluid-fluid levels were assessed. The MR images were classified according to the classification of Mellado and Bencardino.

**Results:**

Most of the lesions were situated peritrochanteric, around the knee or the lower leg. The majority of the lesions had a heterogeneous hypoechoic appearance with septations and intralesional fat globules. On MRI, most of the collections were hypointense on T1-weighted images and hyperintense on T2-weighted images. Half of the collections were encapsulated, and most collections demonstrated septations. The collections were classified as seroma (n = 10), subacute hematoma (n = 2) and chronic organizing hematoma (n = 5).

**Conclusion:**

Ultrasound is the imaging method of choice to diagnose Morel-Lavallée lesions. MRI can be of use in selected cases (extension in different compartments, large collections, superinfection). Characteristic imaging features include a fusiform fluid collection between the subcutaneous fat and the underlying fascia with internal septations and fat globules. On MRI, six types of ML lesion can be differentiated, with the seroma, the subacute hematoma, and the chronic organizing hematoma being the most frequently observed lesions.

## Introduction

In 1863, the French surgeon Victor Auguste François Morel-Lavallée originally described posttraumatic fluid collections dissecting the subcutaneous fat, resulting from soft tissue injury to the proximal thigh [1]. Already many terms have been given to this soft tissue injury, e.g. closed degloving injury, posttraumatic soft tissue cyst, Morel-Lavallée effusion, and chronic expanding hematoma [2,3,4]. These closed degloving injuries have a predilection for the peritrochanteric region and the proximal thigh, where they are specifically referred to as Morel-Lavallée (ML) lesions [5,6]. Similar lesions can be found throughout the entire body however [7].

Severe trauma to the pelvis or thigh, such as motor vehicle accidents or low-velocity crush injuries, may result in a ML lesion [4]. This lesion is often identified within hours to days after the initial trauma, but up to one third of the patients present only months or years after the initial injury, thereby losing the causing link with the initial trauma [8]. ML lesions can occur isolated but may also be associated with an underlying fracture [9,10]. Patients often present with pain and swelling in the affected area and physical examination reveals a soft fluctuant mass with contour deformity [8,11]. If the cutaneous nerves are injured during the shearing injury, a decreased cutaneous sensation of the skin over the degloved area can be observed [2].

**Figure 1:**
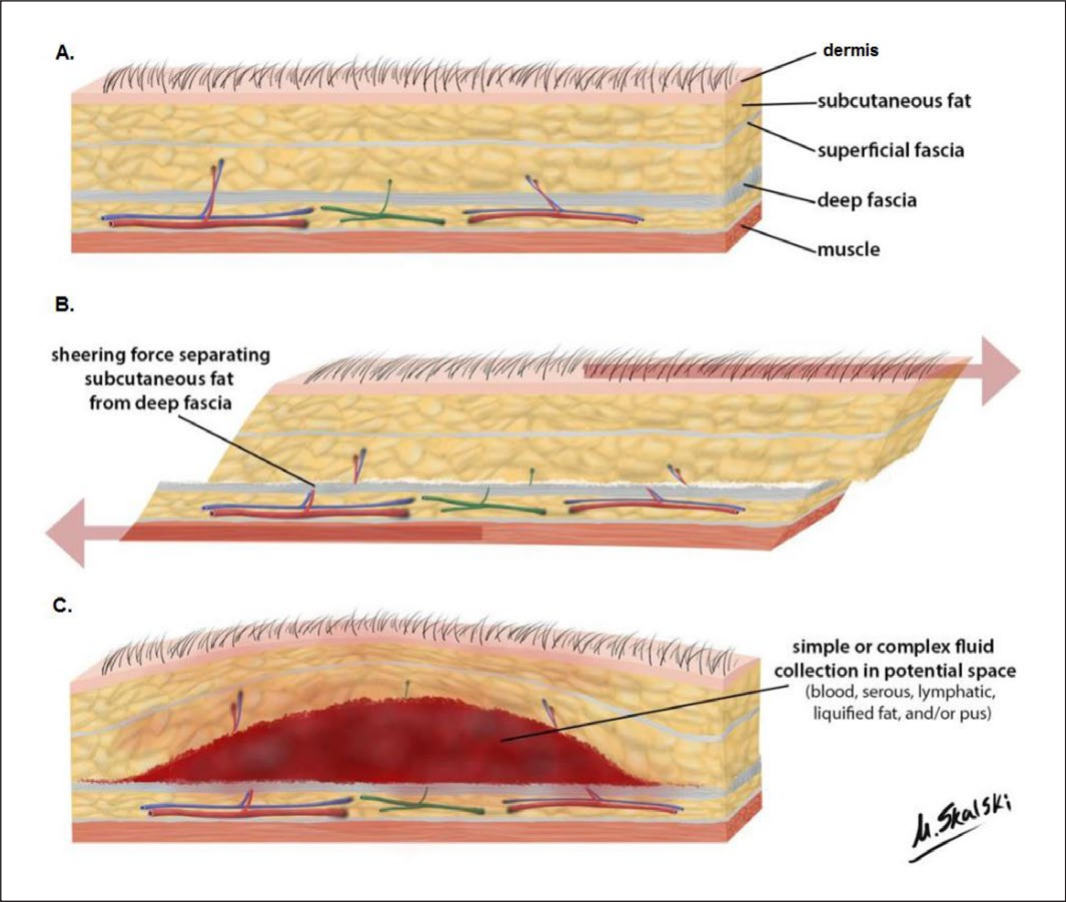
Mechanism of injury. Cross-sectional schematics of: (**A**) Normal layers of tissue from dermis to bone. (**B**) Tangential shearing force cause the relatively mobile dermis and subcutaneous fat to move relative to the fixed underlying fascia, causing disruption of perforating arteries (red), veins (blue), and lymphatics (green). (**C**) Formation of a hemolymphatic collection in the potential space between subcutaneous fat and fascia. Case courtesy of Dr Matt Skalski.

The mechanism of injury is a sudden shearing force that occurs tangential to the fascial planes, causing the somewhat mobile dermis and subcutaneous fat to abruptly move relative to the firmer underlying fascia (Figure 1) [12]. The dissecting trauma can cause disruption of perforating capillaries and lymphatics, and these disrupted capillaries may continuously drain into the perifascial plane, leading to the accumulation of lymph, blood, debris, and fat in the interfascial plane, which in turn leads to the formation of a heterogeneous collection in this potential space [3,6].

The peritrochanteric area is particularly sensitive to this injury due to the increased mobility of the soft tissues in this region, the superficially located bone, a strong underlying fascia lata attaching to the iliotibial band, and a rich vascular plexus piercing the fascia lata [6].

Several forms of subcutaneous fluid collections may result, depending on the severity of the injury, ranging from seroma to hematoma. After some time, the blood is reabsorbed and replaced by serosanguineous fluid [11]. This hemolymphatic collection may continue to enlarge, and patients may present with a large mass, usually located over the external aspect of the thigh. Because of this enlarging nature, these collections may be mistaken as a soft tissue tumour [2,3].

This serosanguineous collection may either spontaneously resolve or undergo a secondary inflammatory reaction with subsequent fibrous capsule formation [2,4,7,9,11,12,13].

**Figure 2:**
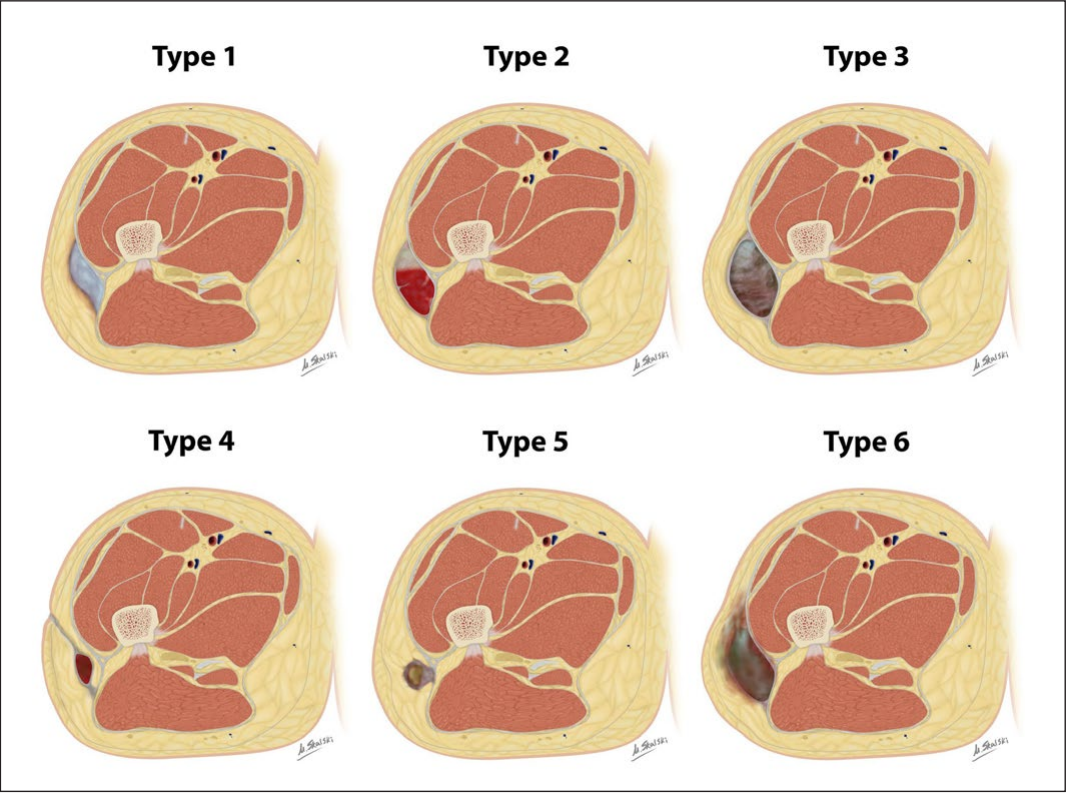
Mellado and Bencardino classification of Morel-Lavallée lesions. Case courtesy of Dr Matt Skalski. (**A**) Type I = seroma, (**B**) Type II = subcatue hematoma, (**C**) Type III = chronic organizing hematoma, (**D**) Type IV = perifascial dissection with closed fatty laceration, (**E**) Type V = perifascial pseudonodular lesion, (**F**) Type VI = infected lesion with or without sinus tract formation, internal septations, and thick enhancing capsule.

In the literature, six types of ML lesions have been described based on the chronicity, tissue composition and lesion appearance on magnetic resonance imaging (MRI) [7]. Mellado and Bencardino proposed this classification based on lesion shape, presence or absence of a capsule, signal intensity (SI) on T1- and T2-weighted images (WI) and enhancement pattern (Figure 2, Table 1) [2,7].

**Table 1:** MR classification of Morel-Lavallée lesions.

	Type I	Type II	Type III	Type IV	Type V	Type VI

	Seroma	Subacute hematoma	Chronic organizing hematoma	Closed laceration	Pseudo-nodular	Infected
Distribution	62%	12%	31%	–	–	–
Morphology	Laminar	Oval	Oval	Linear	Round	Variable sinus tract
T1 signal	Low	High	Intermediate	Low	Variable	Variable
T2 signal	High	High	Heterogeneous	High	Variable	Variable
Capsule	Sometimes	Thin	Thick	Absent	Variable	Thick

The goal of our study was to retrospectively analyse patients with ML lesions on both ultrasound and MRI. We classified the lesions according to the above mentioned classification, and we subsequently briefly reviewed the imaging features of ML lesions.

## Material and Methods

From 2005 to 2016, 31 patients [mean age = 46 years (range 13–85)] were included in this retrospective cohort study. The search terms *Morel-Lavallée* and *closed degloving injury* were inserted in the search engine of the PACS system; the imaging reports and associated MR-images were analysed and 31 patients were included in the study. Fifteen patients had ultrasound examinations available and 16 patients were evaluated with MRI. The location and morphology were analysed for all patients on both imaging modalities:

1. location: greater trochanter/hip, proximal thigh, suprapatellar region of the knee, abdominal wall, periscapular, scalp, arm, hand, lumbosacral region.
2. morphology: fusiform, oval, laminar.

The ultrasound examinations (both report and images) were specifically evaluated for the following characteristics: echogenicity, internal septations, hyperechoic fat globules, compressibility and Doppler signal.

The following characteristics were evaluated on the MR-images: T1- and T2-SI, presence of a (pseudo) capsule, septations or nodules within the collection, internal enhancement (if intravenous contrast was administered), mass-effect and fluid-fluid levels.

The patients with MR-images were classified according to the classification of Mellado and Bencardino, who proposed an MRI classification of ML lesions corresponding to their histopathological stadium [7]. Six categories have been defined, wherein the first three types are the most frequently observed. An essential factor for determining the MRI appearance of the ML lesion is the age of the blood within the collection [11].

### MRI classification

A *type I ML lesion* is a seroma which exhibits fluid-like signal characteristics on MRI (Figure 2A). This type appears homogeneously hypointense on T1-WI and hyperintense on T2-WI, respective to muscle [13]. This lesion can be found in both acute or chronic settings, and is most often not encapsulated [7].

A *type II lesion* is a subacute hematoma and exhibits a predominantly homogeneous hyperintense SI on both T1- and T2-WI (Figure 2B) [13,14]. The high SI on T1-WI is caused by the presence of methemoglobin; the methemoglobin is first observed in the periphery of early subacute hematomas and progressively acquires a more homogeneous distribution [7,15]. Subacute hematomas often have a hemosiderin-rich hypointense capsule on both T1- and T2-WI. In some subacute hematomas, internal inhomogeneity is observed caused by entrapped fat globules, internal septations or fluid-fluid levels [15]. The presence of capillaries within the lesion can lead to patchy internal enhancement, falsely positive leading to the diagnosis of a soft tissue tumour. These subacute hematomas can further be subdivided in early and late subacute hematomas. The early ones tend to be more homogeneous whereas the late ones are more often heterogeneous with internal septations and debris and develop a fibrous capsule [2].

*Type III ML lesions* are chronic organizing hematomas that demonstrate hypo- or intermediate SI on T1-WI and heterogeneous intermediate to hyperintense SI on T2-WI (Figure 2C). The heterogeneous signal is due to the presence of hemosiderin, granulation tissue, necrotic debris, fibrin, and blood clots [7]. Patchy internal and peripheral enhancement can be seen on post-contrast MR [13], due to neovascularization and granulation tissue. These lesions can be surrounded by a hemosiderin-rich fibrous capsule [2].

The other three categories are longstanding ML lesions and they may demonstrate more atypical MRI features. A *type IV ML lesion* represents a closed fatty tissue laceration in combination with a perifascial dissection, and can be associated with or without a serous/hemorrhagic collection (Figure 2D) [7]. The collection has low SI on T1-WI and high SI on T2-WI, is not encapsulated and demonstrates variable enhancement [2].

A *type V ML lesion* has a small, rounded pseudonodular appearance and is perifascially located (Figure 2E). They occasionally can demonstrate irregular peripheral enhancement and skin retraction [7].

A *type VI lesion* is an infected ML lesion, often with a thick enhancing capsule, internal septations, inflammation of the adjacent fatty tissue and fascia, peripheral fluid leakage, and sometimes an associated sinus tract (Figure 2F) [7].

## Results

### Location

All collections were located between the subcutaneous fat and the adjacent fascia. About one-third of the lesions were situated about the knee (10/31 = 32%). Other locations were the anterolateral compartment of the lower leg (7/31 = 23%), greater trochanter/hip (8/31 = 26%), gluteal (1/31 = 3%), ankle (2/31 = 6%), upper arm (1/31 = 3%) and lumbosacral region (2/31 = 6%). The predominant morphology was fusiform (26/31 = 84%), and oval collections were only seen to a minor extent (5/31 = 16%). Only 3 of the injuries extended in different compartments and demonstrated underlying muscle contusion.

### Ultrasound

**Figure 3:**
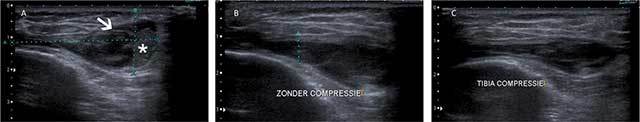
Ultrasound images of a hypoechoic heterogeneous collection in the lower leg/calf (**A**) with internal septations (white arrow) and fat globules (asterisk). The collection is not compressible **(B–C)**.

The majority of the collections demonstrated a heterogeneous hypoechoic ultrasound appearance with intralesional septations (13/15 = 87%) and/or hyperechoic fat globules (10/15 = 67%) (Figure 3). Only a minority of the injuries (2/15 = 13%) were homogeneously anechoic. All but one of the collections were compressible and none demonstrated internal vascular flow.

### MRI

**Figure 4:**
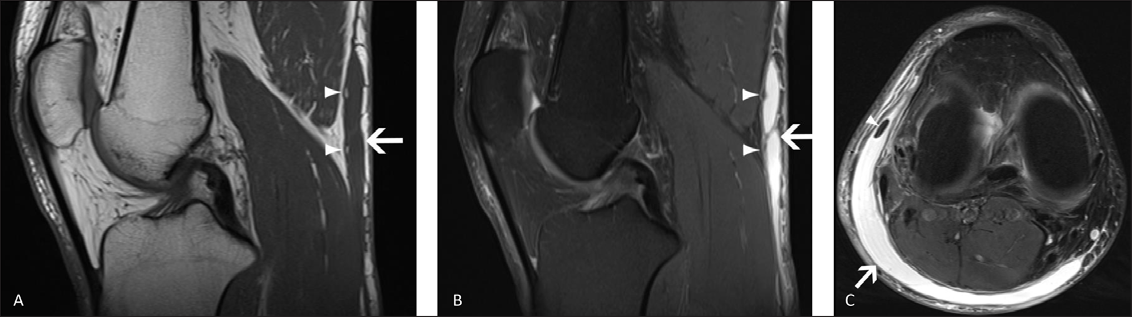
A 42-year-old man with a marked swelling of the lower leg after direct impact to the calf due to a motor vehicle accident. Type I lesion: seroma. Sagittal T1 (**A**), sagittal PD FS (**B**) and axial T2 (**C**) MR images demonstrate an oval smooth collection (white arrows) between the subcutaneous fat and the fascia of the calf muscle, demonstrating low SI on T1- and high SI on T2-WI. A small focal area of isointense signal compared to subcutaneous fat was seen within the lesion (white arrowheads), corresponding with an internal fat globule.

**Figure 5:**
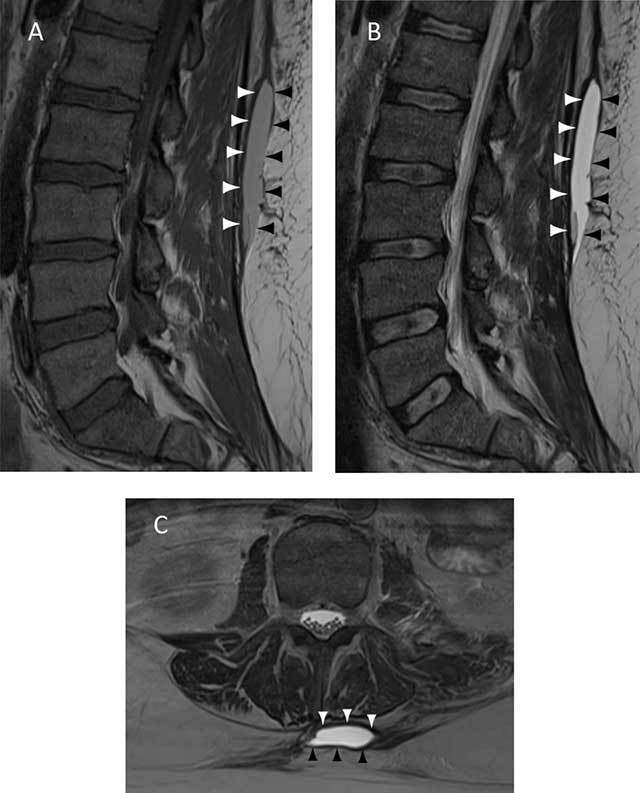
A 51-year-old man with low back swelling status post trauma. Type II lesion: subacute hematoma of the lumbar region. Sagittal T1 (**A**), sagittal T2 (**B**) and axial T2 (**C**) MR images of the lumbar region demonstrate a collection between the thoracolumbar fascia (white arrowheads) and the overlying subcutaneous fat and superficial fascia (black arrowheads). This subacute hematoma has a homogeneously high SI on T1-WI relative to muscle, consistent with methemoglobin.

**Figure 6:**
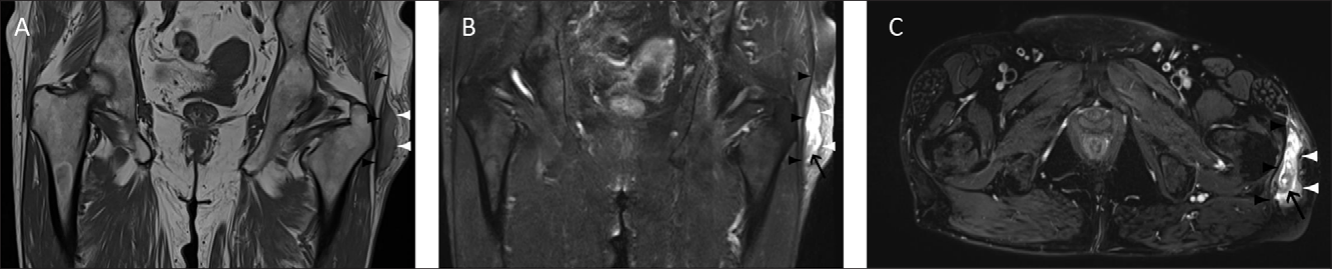
A 58-year-old man presenting with a left hip mass. Type III Morel-Lavallée lesion: chronic organizing hematoma. Coronal T1 (**A**), coronal PD FS (**B**), and axial PD FS (**C**) MR images of the left hip show a complex mass between the fascia lata (white arrowheads) and the iliotibial tract (black arrowheads). The mass has an intermediate SI on T1-WI and high SI on T2-WI. The inhomogeneity of the collection represents the separation of blood products and debris (black arrow). A peripheral, thickened, low SI capsule is seen.

Most of the collections were of hypointense SI on T1-WI (8/16 = 50% hypointense, 7/16 = 44% isointense, 2/16 = 12% hyperintense) and all cases were hyperintense on T2-WI. More than half of the collections were encapsulated (11/16 = 68%) and demonstrated internal septations (9/16 = 56%). Fat globules were present in fifty percent of the cases (8/16). None of the collections showed enhancement (note that intravenous gadolinium contrast was only administered in three patients). Three collections revealed mass-effect on surrounding structures and fluid-fluid levels were present in one patient. The collections were classified according to Mellado and Bencardino as seroma (type I; 10/16 = 62%) (Figure 4), subacute hematoma (type II; 2/16 = 12%) (Figure 5) and chronic organizing hematoma (type III; 5/16 = 31%) (Figure 6) (Table 1).

## Discussion

Morel-Lavallée lesions are most commonly located within the soft tissues superficial to the greater trochanter. Other commonly reported locations are the thigh or about the knee [8,9,11,16]. This distribution is similar with our series (knee 32%, greater trochanter 29%, anterolateral compartment lower leg 23%). Less commonly reported locations include the lumbosacral region, the abdominal wall (usually related to prior abdominoplasty), the calf, periscapular region, scalp, arm, and hand [2,11,17], which is in line with our observations. The following locations were reported in a literature review of 204 ML lesions from 29 articles: greater trochanter 37%; thigh 20.1%; pelvis (other than trochanteric area) 18.6%; knee 15.7%; lumbosacral 3.4%; abdominal 1.5%; calf/lower leg 1.5%; head (subgaleal) 0.5%; and not specified 2.0% [18].

ML lesions most often appear as well-defined oval or fusiform cystic structures with differing degrees of internal complexity on all imaging modalities [6]. In our study, the predominant morphology was fusiform (26/31 = 84%) and oval collections were only seen to a minor extent (5/31 = 16%). ML lesions may contain blood, lymph, and debris. Depending on the age of the internal blood products, they can have a variable imaging appearance. Ultrasound is the imaging modality of choice to investigate patients with clinical suspicion of a ML lesion. They appear as fluid collections, often with heterogeneous echogenicity. The echogenicity depends on the degradation stage of the blood products [19,20,21,22]; acute and subacute (<1 month) lesions will appear heterogeneous with irregular margins and lobular shape. The more acute the hematoma, the more hyperechoic the collection will appear. Over time the blood products will liquefy and become more hypoechoic [6]. Chronic lesions (> 18 months) are more often homogeneous with smooth margins and flat or fusiform shape [21]. Generally, ML lesions are compressible with the ultrasound transducer and demonstrate no flow on colour Doppler. Underlying muscle contusion or laceration can sometimes be demonstrated with ultrasound [2,23]. Neal, et al. investigated the ultrasound characteristics of 21 ML lesions located around the hip and knee [21]. The majority demonstrated a heterogeneous hypoechoic appearance, with less than 1/3 being purely anechoic. This is similar to our observations (87% heterogeneous hypoechoic; 13% anechoic). All collections were compressible and demonstrated no vascularity, similar to the observations in our series. Neal, et al. believes that the presence of internal areas of nodular echogenicity correspond with areas of disrupted, sheared subdermal fat globules and secondary necrosis [21]. These intralesional fat globules may be seen as small hyperechoic foci [12], as we observed in 67% of the patients. Their presence is not pathognomonic, but it is believed that this imaging feature can aid to characterize the collection as an ML lesion [4,19,21]. Some lesions may contain internal septations or a fluid-fluid level.

MRI demonstrates exquisite soft-tissue contrast and has the ability to gauge the chronicity of the hematoma [6]. It is the modality of choice when a more global overview of the lesion is required. It provides the clinician with a more precise assessment of the lesion’s extent (e.g. underlying muscle contusion) and morphology, particularly in large lesions. The SI of the lesion varies according to the stage of degradation of the internal blood products. Each stage has its corresponding SI characteristics [12].

The differential diagnosis of ML includes soft tissue masses and other posttraumatic collections. In the group of the soft tissue masses, sarcomas are the most important differential and may cause a diagnostic dilemma. A soft tissue sarcoma may mimic a type I or III ML lesion. A distinguishing feature between sarcomas and ML lesions are intralesional areas of marked homogeneous enhancement, exclusively seen in sarcomas [2,24].

Another differential diagnosis is bursitis; the location is usually the clue to the correct diagnosis, e.g. location of a fluid collection in the prepatellar region is mostly due to bursitis. However, chronic hemorrhagic bursitis may mimic a type III ML lesion [2,24]. A prepatellar bursitis however does not extend beyond the mid-coronal plane medially or laterally or to the mid-thigh proximally, whereas prepatellar ML lesions often extend medially and laterally at the mid- to distal thigh, a location distinct from the prepatellar bursa [25,26].

Other differential diagnostic dilemmas are posttraumatic collections such as fat necrosis and coagulopathy-related hematomas [2]. The MR appearance of fat necrosis depends on the timing after trauma. It can appear spiculated, globular or more laminar [27], and sometimes may mimic a type IV (pseudonodular) ML lesion [7].

A pseudolipoma can develop posttraumatic after blunt trauma or iatrogenic after surgery and is often preceded by a hematoma [7,19]. On MRI it presents as a non-encapsulated subcutaneous lipomatous mass without contrast enhancement [28].

Coagulopathy-related hematomas are often out of proportion to the degree of trauma, or may occur even in an atraumatic setting. Patient history and laboratory test are most of the time sufficient to lead to a correct diagnosis [7].

Treatment depends on the stadium of the ML lesion. Management options include compression banding, aspiration, or incision and evacuation, with or without injection of sclerosing agents [2,29].

In acute posttraumatic ML lesions, a conservative approach with aspiration and compression bandages first may be attempted. If the lesion does not resolve it may require percutaneous drainage. If the drainage is not sufficient, an open debridement with resection of the necrotic tissue is performed to avoid risk of infection [2,7].

In chronic lesions, percutaneous drainage with sclerotherapy can be attempted. In lesions in which a peripheral capsule has formed, capsule resection may be required in order to prevent re-accumulation of hemolymphatic fluid [12].

## Conclusion

Ultrasound is the imaging method of choice to diagnose Morel-Lavallée lesions. MRI can be useful in selected cases (extension in different compartments, large collections, suspicion of superinfection, differential diagnosis with soft tissue sarcoma). Characteristic imaging features include a fusiform fluid collection with septations and fat globules between the subcutaneous tissues and the underlying fascia.

On MRI, six types of ML lesion can be differentiated, with the seroma, the subacute hematoma, and the chronic organizing hematoma being the most frequently observed lesions. Knowledge of the common locations, the specific location between the subcutaneous tissue and the fascia, the imaging signs, and knowledge of the classification system may all be helpful in the differential diagnosis with other fluid-filled collections in the soft tissues.
